# Limits of ZnO Electrodeposition in Mesoporous Tin Doped Indium Oxide Films in View of Application in Dye-Sensitized Solar Cells

**DOI:** 10.3390/ma7043291

**Published:** 2014-04-23

**Authors:** Christian Dunkel, Till von Graberg, Bernd M. Smarsly, Torsten Oekermann, Michael Wark

**Affiliations:** 1 Institute of Physical Chemistry and Electrochemistry, Gottfried Wilhelm Leibniz University Hannover, Callinstrasse 3a, 30167 Hannover, Germany; E-Mails: torsten.oekermann@friwo-batterien.de (T.O.); michael.wark@uni-oldenburg.de (M.W.); 2 Institute for Chemistry—Photocatalysis and sustainable feedstock utilization, Carl-von-Ossietzky University Oldenburg, Carl-von-Ossietzky Strasse 9-11, 26129 Oldenburg, Germany; 3 Wiley-VCH Verlag GmbH & Co. KGaA, Boschstrasse 12, 69469 Weinheim, Germany; E-Mail: tgraberg@wiley.com; 4 Institute of Physical Chemistry, University of Giessen, Heinrich-Buff-Ring 58, 35392 Giessen, Germany; E-Mail: bernd.smarsly@phys.chemie.uni-giessen.de; 5 Friemann & Wolf Batterietechnik GmbH, 63654 Büdingen, Germany

**Keywords:** mesoporous TCO, zinc oxide, electrodeposition, dye-sensitized solar cells (DSSC), indium tin oxide (ITO)

## Abstract

Well-ordered 3D mesoporous indium tin oxide (ITO) films obtained by a templated sol-gel route are discussed as conductive porous current collectors. This paper explores the use of such films modified by electrochemical deposition of zinc oxide (ZnO) on the pore walls to improve the electron transport in dye-sensitized solar cells (DSSCs). Mesoporous ITO film were dip-coated with pore sizes of 20–25 nm and 40–45 nm employing novel poly(isobutylene)-b-poly(ethylene oxide) block copolymers as structure-directors. After electrochemical deposition of ZnO and sensitization with the indoline dye D149 the films were tested as photoanodes in DSSCs. Short ZnO deposition times led to strong back reaction of photogenerated electrons from non-covered ITO to the electrolyte. ITO films with larger pores enabled longer ZnO deposition times before pore blocking occurred, resulting in higher efficiencies, which could be further increased by using thicker ITO films consisting of five layers, but were still lower compared to nanoporous ZnO films electrodeposited on flat ITO. The major factors that currently limit the application are the still low thickness of the mesoporous ITO films, too small pore sizes and non-ideal geometries that do not allow obtaining full coverage of the ITO surface with ZnO before pore blocking occurs.

## 1. Introduction

Transparent conductive oxide (TCO) films have received significant attention in the past few years, as they are applicable as transparent electrodes in liquid crystal displays [1] and organic light-emitting diodes [2]. The most widely used TCO material is tin-doped indium oxide (indium-tin-oxide, ITO), which has high electrical conductivity and transparency [3,4]. Other promising TCOs include fluorine-doped tin oxide (FTO) [5], antimony-doped tin oxide (ATO) [6], aluminium-doped zinc oxide (AZO) [7] and zinc-indium-tin oxide (ZITO) [8].

In some examples, it was shown that especially TCO films with defined mesoporosity could be of interest as e.g., sensors [9], electrodes in Li batteries [10], electrochromic devices [11] or for bioelectronics [12]. Such mesostructured TCO films have a significantly higher specific surface area than flat ITO films, allowing functionalization with dyes, electrochemically and photoelectrically active species as well as immobilization of redox enzymes within the mesoporous framework [11–13].

The first attempts applying template-assisted sol-gel syntheses provided only porous ITO powders with quite high resistivity [14] or irregular porosity resulting from the loose packing of crystallites [15]. In an initial work successfully resulting in ordered mesoporous ITO films the commercially non-available KLE (H(CH_2_CH_2_CH_2_(CH)CH_2_CH_3_)_89_(OCH_2_-CH_2_)_79_OH) block copolymer was employed to prepare 3D arrangements of spherical mesopores of about 13 nm diameter. However, these mesopores shrank by up to 50% (to about 7 nm) in the *z*-direction (normal to the film plane) upon annealing [13]. In a recent paper Liu *et al.* [16] report on the synthesis of nanostructured transparent conducting ITO materials based on controlled self-assembly of ultra-small indium tin hydroxide nanoparticles. Nanosized, nearly spherical and highly dispersible ITO nanoparticles were assembled into regular mesoporous architectures directed by a commercially available Pluronic^®^ polymer. After mild heat treatment at 300 °C the resulting ITO layers featured a regular mesoporosity with a mesostructure periodicity of about 13 ± 2 nm, high surface area of 190 m^2^·cm^−3^, porosity of 44% and electrical conductivity up to 9.5 S·cm^−1^.

Electrochemical deposition of ZnO is a well-studied method to obtain photoanodes for application in DSSC [17]. Since the necessary Zn^2+^ and OH^−^ ions can penetrate the ITO pores the formation of a layer consisting of ZnO nanoparticles should be possible. DSSCs commonly apply films of dye-sensitized semiconductor nanoparticles as light absorbing layers. A high surface area of the nanoparticle film is needed in order to achieve a sufficiently high efficiency, since only a monolayer of dye molecules directly adsorbed on the surface can contribute to the photocurrent [18]. Thus a high surface area is needed which requires a high thickness of a porous film as well as efficient electron transport through the film. However, electron diffusion in a porous semiconductor electrode is usually a slow process, which is further slowed down due to grain boundaries in case of nanoparticular films [19]. To shorten the electron transport time through the porous light absorbing layer, Zaban *et al.* [20,21] proposed a concept of a collector-shell electrode, where a nanoparticular TCO film of (ITO or ATO) is coated with a thin layer of the semiconducting material. However, this approach still suffers from grain boundaries between the TCO nanoparticles as well as from irregular pore shapes, narrow pore openings and a wide pore size distribution, ranging from micro- to macropores complicating the deposition of a homogeneous thin semiconducting layer on the pore walls as well as the wetting with the redox electrolyte.

A fully accessible and ordered 3D mesoporous film of a transparent conducting oxide was discussed to be advantageous [13]. In this paper, the principle feasibility to deposit zinc oxide (ZnO) electrochemically into mesoporous TCO layers was already demonstrated for KLE-templated ITO films. However, by using the dye eosin Y as sensitizer the resulting photocurrents and photovoltages were extremely low (*J*_SC_ = 73 μA·cm^−3^, *V*_OC_ = 286 mV), in part due to the rather narrow absorption band of the eosin Y. In order to obtain reasonable efficiencies in DSSCs, the ZnO layer has to be fully compact, so that no back reaction of photogenerated electrons from bare ITO surface to the redox electrolyte can occur. Furthermore, the pores have to remain fully accessible in order to enable unhindered dye adsorption and unhindered ion transport in the working cell.

The use of poly(isobutylene)-b-poly(ethylene oxide) (PIB-PEO) block copolymers as structure-directing agents allows to generate 3D ordered mesoporous ITO films with pore sizes larger than 20 nm. The actual pore size depends on the lengths of the PIB and the PEO blocks, which is 50 (PIB) and 45 (PEO) for PIB-PEO 3000 or 353 (PIB) and 454 (PEO) for PIB-PEO 20000 leading to spherical mesopores with sizes of 20–25 nm and 40–45 nm, respectively. The dc-conductivity of these films was reported to be 0.6 ± 0.3 S·cm^−1^ and 1.9 ± 0.3 S·cm^−1^ [11]. Although the conductivity of these films is lower than those reported for the Pluronic^®^ templated ones, the much higher pore diameter should be beneficial for the electrochemical deposition of ZnO in the ITO pores.

In this study the effects of different pore sizes of the mesoporous ITO, its pre-treatment before the ZnO electrodeposition and the ZnO deposition time on the DSSC efficiency were analysed. Additionally, the thickness of the mesoporous ITO films was increased by stacking multiple ITO layers. Instead of eosin Y used in the initial study [13] a state-of-the-art sensitizer, the indoline dye D149, was used in the DSSC. This dye is especially suited for electrodeposited ZnO and performs higher than the N719 dye [22] which is commonly used in DSSCs with nanoparticulate titanium dioxide films. Thus, our study provides a comprehensive investigation on the potential use of mesoporous TCO films with thin layers of ZnO electrodeposited on the pore walls as electrode material in DSSCs.

## 2. Results and Discussion

### 2.1. Single Layered Mesoporous ITO Films

#### 2.1.1. Summary of Mesoporous ITO Film Characteristics

**Figure 1.**
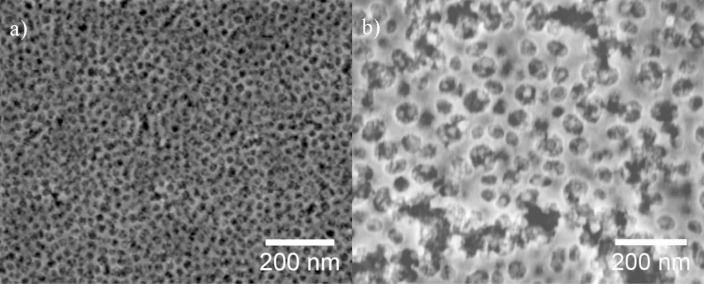
SEM images of mesoporous indium tin oxide (ITO) films templated with the block copolymers PIB-PEO 3000 (**a**) and PIB-PEO 20000 (**b**).

Figure 1 shows SEM images of mesoporous ITO films which were templated using the block copolymer PIB-PEO 3000 (a) and PIB-PEO 20000 (b). The films prepared with PIB-PEO 3000 (Figure 1a) have a very homogeneous surface almost without irregularities. In top view the pores are circular (note that in cross sections due to some shrinkage they are ellipsoidal, as can be seen in Chapter 2.1.3, Figure 4) and exhibit a diameter of about 20 nm, and the pore walls have a thickness of 8 to 10 nm. In comparison, the surface of the film prepared with PIB-PEO 20000 (Figure 1b) is less homogeneous. The pores of these films are circular in top view, possessing a diameter between 35 and 45 nm, while the pore walls have a thickness of typically 12 to 18 nm, which however increases at several places up to 50 nm. The quantitative analysis of the EDX spectra by the Cliff-Lorimer ratio technique [23] gave a tin content on the metal content (Sn/(In + Sn)) of 8.2% for PIB–PEO 3000 and 10.8% for PIB-PEO 20000 templated ITO films, which is, taking the measurement and weighing error into account, in good agreement with the weight in the sol. BET surface and conductivity were analyzed in an earlier study by von Graberg *et al.* [11]. In brief: Kr adsorption measurements at 77 K revealed a BET surface of 410 m^2^·cm^−3^ for a 110 ± 10 nm thick PIB-PEO 3000 templated film and 496 m^2^·cm^−3^ for a 145 ± 20 nm thick PIB-PEO 20000 templated film. Both kinds of films are highly crystalline and possess a dc-conductivity of 0.6 ± 0.2 S·cm^−1^ (PIB-PEO 3000) and 1.9 ± 0.3 S·cm^−1^ (PIB-PEO 20000), the larger value of the latter film being explained by the thicker pore walls. The transparency of the films is over 80% and equal to the ITO substrate (see supplementary information Figure S1).

#### 2.1.2. Influence of the Pre-Treatment of the Mesoporous ITO

To increase the conductivity, the as-prepared ITO films were reduced by N_2_, which causes an increase in the number of oxygen vacancies and thereby an increased doping level and conductivity [24]. Treatment in an N_2_ atmosphere at 300 °C for 2 h led to a sheet resistance of ~90 kΩ/square, compared to a sheet resistance of ~360 kΩ/square of the as-prepared films (before N_2_ treatment). The opposite effect was found by ageing the films in air for 100 days at room temperature leading to an increase of the sheet resistance to ~1200 kΩ/square (all values measured at mesoporous ITO films on non-conductive glass by 4-point probing).

**Figure 2.**
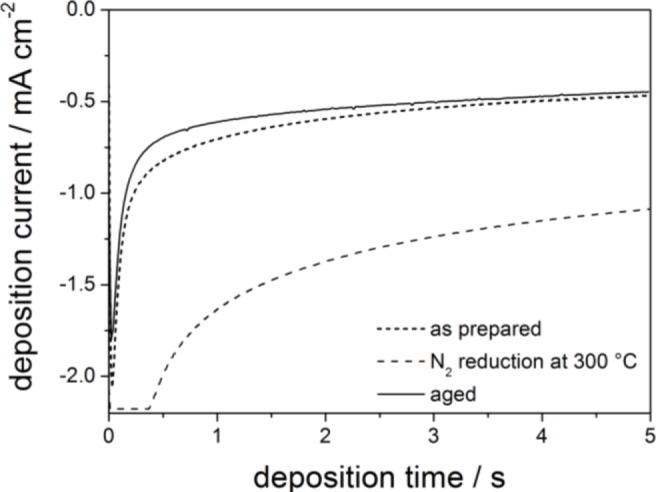
Current transients during electrodeposition of ZnO on as prepared (…), N_2_ reduced (- -) and aged (—) PIB-PEO 3000 templated ITO films at a deposition potential of −0.91 V *vs.* Ag/AgCl.

The current transients measured during electrodeposition of ZnO for 5 s in the three different conductive films are shown in Figure 2. The highest current density is observed with the reduced substrate, which is expected due to its higher conductivity compared to the other two samples. Note that a maximum current density of 6.5 mA (substrate area of 3 cm^−2^) was set at the potentiostat, leading to the current plateau at the beginning of the deposition curve. Ageing in air slightly decreases the electrodeposition current compared to the as-deposited film, which is consistent with the decreased conductivity.

**Figure 3.**
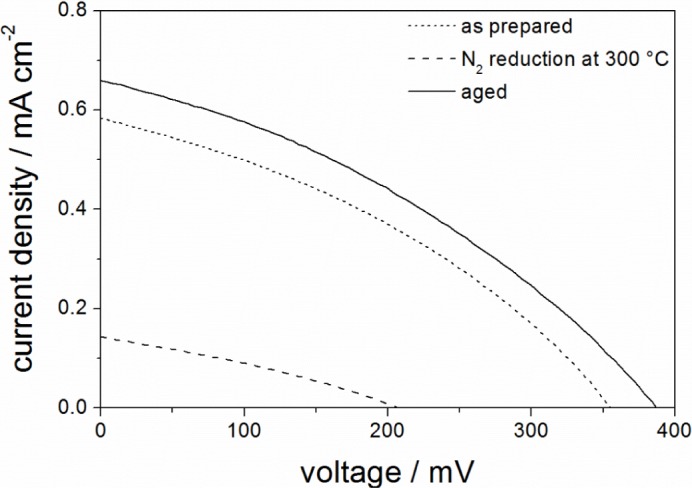
*J*-*V*-curves of dye-sensitized solar cells (DSSC) with D149-sensitized ZnO deposited into as prepared (…), N_2_ reduced (- -) and aged (—) mesoporous ITO films prepared with PIB-PEO 3000.

Figure 3 shows the *J*-*V*-curves of all three films measured under illumination after sensitization of the electrodeposited ZnO with D149 dye. It is clearly observed that lower conductivities of the ITO lead to higher *J*_SC_ (short-circuit current) and *V*_OC_ (open-circuit voltage) values. As pointed out in the introduction, incomplete coverage of the ITO surface, leading to accelerated back transfer of photogenerated electrons to the redox electrolyte, was another suspicious reason for the very low efficiencies obtained in the previous study [13]. Although the larger pores of the ITO films used here should enable a higher coverage, some remaining direct contact between the ITO and the redox electrolyte is still likely. This means that a very high conductivity of the ITO would improve the electron transport through the film, however, on the other hand, could also lead to more electron losses by back reaction. The obtained results indicate that the increased back reaction weighs more than the faster electron transport. Thus, all further investigations in this study were therefore performed using aged mesoporous ITO films.

The incomplete coating also indicates that only single ZnO nanocrystals were formed on the inner ITO surface instead of a complete ZnO layer. However, the question remains why the much higher amount of ZnO electrodeposited in the reduced ITO film does not suppress back reaction efficiently. A possible explanation is that the electrochemical deposition of the ZnO preferably occurs at the pore necks, where the electrical field strength will reach its highest value due to the high curvature in the network and through which the educts for ZnO formation have to pass to reach the pores. Therewith, accelerated ZnO formation may lead to an early blocking of the pore openings, hindering further ZnO deposition inside the pores. Thus ITO films providing straight channel pores will probably perform better; however, for such systems until now no suitable templates are available.

#### 2.1.3. Investigation of the ZnO Electrodeposition by STEM and EDXS Analysis

**Figure 4.**
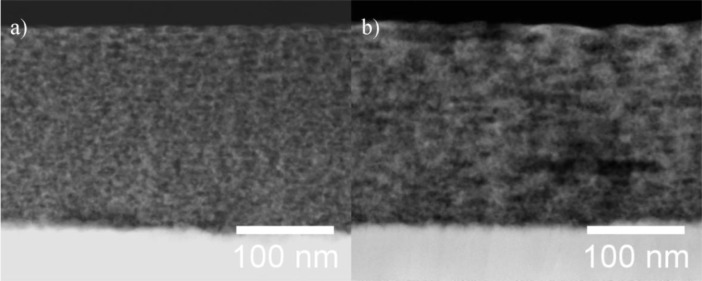
STEM dark field image of mesoporous ITO films prepared with the block copolymer PIB-PEO 3000 (**a**) and PIB-PEO 20000 (**b**) in cross section after 10 s of ZnO electrodeposition. In both cases no ZnO crystals are observed on top of the films.

STEM/EDXS analysis was performed on aged films of both types of PIB-PEO templated ITO films after electrodeposition of ZnO (Figure 4). In both cases the thickness of the mesoporous ITO films is about 200 nm, and no ZnO crystals are visible on top of the ITO films. However, the obtainable resolution was not sufficient to observe ZnO crystals within the nanoporous system.

**Figure 5.**
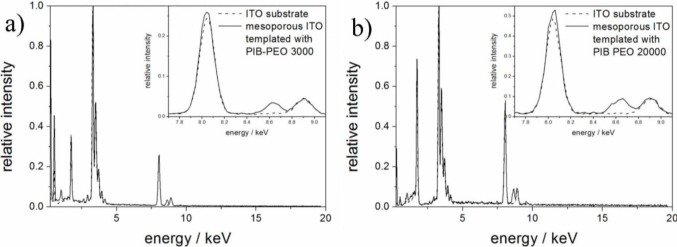
Comparison of the EDX spectra extracted of compact ITO layer and the mesoporous part of the ITO film prepared with PIB-PEO 3000 (**a**) and PIB-PEO 20000 (**b**) after 10 s of ZnO deposition, respectively. The Zn-K_α_ signal is visible at 8.64 eV.

ZnO electrodeposited in the pores of the ITO films could be verified by EDX spectra (Figure 5) performed on the cross sections shown in Figure 4. While the emissions of copper at 8.04 and 8.90 eV are a consequence of the measurement technique and are not related to the sample (X-ray fluorescence of the high resolution pole piece), both films also exhibit the characteristic Zn-K_α_ emission at 8.64 eV. The quantitative analysis of the EDX spectra from the mesoporous parts of the substrate revealed Zn/(In + Sn + Zn) ratios of 2.7% for PIB-PEO 3000 templated films and 6.5% for PIB-PEO 20000 templated films, respectively. The higher Zn content of the latter film can be expected due to the two times larger pores of the PIB-PEO 20000 templated films compared to PIB-PEO 3000 templated films, enabling the deposition of more zinc in the pores of the mesoporous ITO before significant blockage of the pore mouths occurs.

#### 2.1.4. Influence of the Duration of ZnO Electrodeposition on DSSC Characteristics

**Figure 6.**
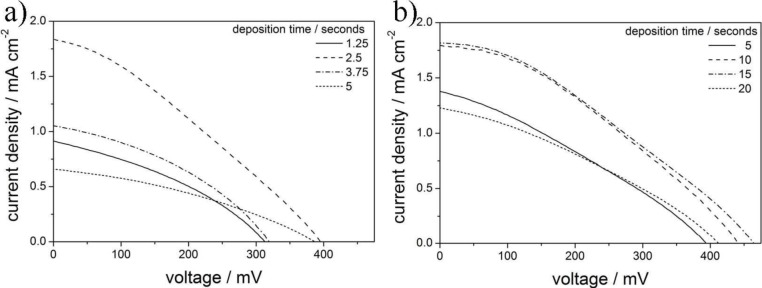
*J*-*V*-curves of D149-sensitized ZnO deposited into aged mesoporous ITO prepared with PIB-PEO 3000 (**a**) and PIB-PEO 20000 (**b**) for different ZnO deposition times.

Figure 6 shows a comparison of *J*-*V*-curves of DSSCs based on aged mesoporous ITO films templated with PIB-PEO 3000 (Figure 6a) and PIB-PEO 20000 (Figure 6b) containing ZnO electrodeposited at different deposition times. Note that the fill factor *FF* of all cells was about ~0.3 and the solar to electrical power conversion efficiency η = *V*_OC_·*J*_SC_·*FF*/*i*_0_ (*i*_0_ being the power of the incident light per cm^2^) follows the same trend as *J*_SC_. The open-circuit voltage *V*_OC_ reaches maximum values of 395 mV for PIB-PEO 3000 templated films and 463 mV for PIB-PEO 20000 templated films. These values are still lower than those of a porous ZnO film on flat ITO (691 mV) [17], indicating the presence of increased electron back reaction from parts of the ITO surface not being covered with ZnO to the electrolyte in the pores. The data also indicate that a better coverage of the ITO walls is achieved in case of the PIB-PEO 20000 templated films, leading to a higher *V*_OC_ value. Interestingly, the dependence of the short-circuit current density *J*_SC_ on the electrodeposition time appears to be quite different for the two kinds of ITO films. For the PIB-PEO 3000 templated films, it reaches a sharp maximum at 2.5 s, followed by a sharp decrease down to *ca.* ¼ of the maximum value at 7.5 s. This peculiar behaviour indicates blocking of the pores for deposition times > 2.5 s. In case of the PIB-PEO 20000 templated films, featuring larger pores, much more ZnO can be deposited before pore blocking becomes an issue, which is seen in the fact that *J*_SC_ only drops for deposition times > 15 s. This higher amount of deposited ZnO results in less recombination and therefore in a higher *V*_OC_ value, eventually leading to a higher maximum efficiency η of 0.28% for the PIB-PEO 20000 templated films, compared to 0.22% for the PIB-PEO 3000 templated films.

To fully overcome the problem of bare ITO being present in the pores, a further enlargement of the pore diameters would be needed to allow an even longer ZnO deposition without pore blocking. However, the benefits of the larger amount of ZnO would be partially or fully offset by the smaller overall surface area of a film with larger pores, so that a careful optimization of the pore size would be needed.

In comparison to DSSCs employing pure nanoporous ZnO films electrodeposited on flat ITO substrates using eosin Y as pore-generating additive, where *J*_SC_ values of up to 12.2 mA·cm^−2^ are reached with the same sensitizer [17], the *J*_SC_ value of 1.9 mA·cm^−2^ achieved in this study appears to be rather low at first view. However, it has to be taken into account, that the higher efficiency of the pure ZnO film is achieved with a much higher film thickness of about 8 μm. Thus, the mesoporous ITO films with an overall thickness of only ca. 200 nm already generates 15.6% of the current density at only 2.5% of the thickness. Pauporté and Rathouský found a specific surface area of 180 cm^2^·cm^−2^ for a ~2.2 μm thick ZnO film electrodeposited with eosin Y as template [25]. This would correspond to a surface area of 82 m^2^·cm^−3^, being only 1/6 of that of the mesoporous ITO films (496 m^2^·cm^−3^) [11]. As result of that comparison it can be concluded that the current density in fact benefits from the high surface area of the mesoporous ITO films.

### 2.2. Five Layer Mesoporous ITO Films Prepared with PIB-PEO 20000

#### 2.2.1. Investigation of the ZnO Electrodeposition by TEM and EDXS Analysis

**Figure 7.**
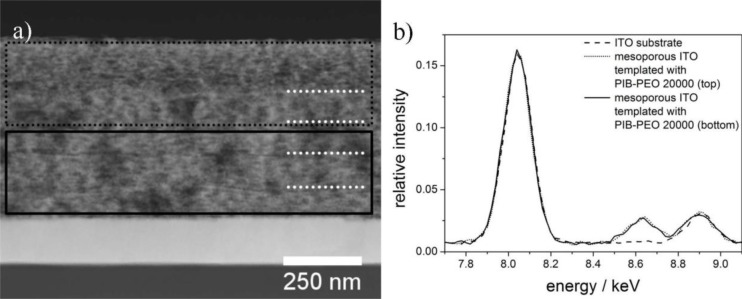
(**a**) Scanning mode (STEM) dark field image of a five-layer mesoporous ITO film prepared with the block copolymer PIB-PEO 20000 in cross section), the dotted and solid boxes show the area from which the EDX spectra were taken, the white dotted lines point out the interfaces between the mesoporous ITO layers; (**b**) EDX spectra of the compact ITO layer and the top and bottom parts of the five-layer mesoporous ITO film after 10 s of ZnO deposition. The Zn-K_α_ signal is visible at 8.64 eV.

In order to increase the overall dye load, the thickness of the mesoporous ITO layers prepared with PIB-PEO 20000, which exhibited higher efficiencies as monolayers, was increased by consecutively dip-coating five layers on the same substrate with a pre-condensation step after each dip-coating process (see experimental part for details). An STEM image of such a film is shown in cross section view in Figure 7a, the white dotted lines on the right side in the image point out the interfaces between the individual layers of the multilayer film. The thickness of the film is about 550 nm, which is, however, only 2.75 times the thickness of a single layered film. This is clearly due to the lower thickness of the first 4 layers, each being about 100 nm thick compared to 150 nm for the top layer. They probably were partly dissolved during the dip-coating process of the respective next layer, leading to a slightly changed structure being less porous than the top layer. In the TEM images the bottom layers appear brighter than the top layer, indicating that they are more dense. In Figure 7b the EDX spectra of the compact ITO layer and the top and bottom parts of the mesoporous ITO are shown. Since the signal intensities of Zn in the top and bottom parts of the porous film are almost identical, it can be concluded that ZnO was homogeneously deposited throughout the multilayered mesoporous ITO film, proving the unhindered accessibility of the entire porous system. The quantitative analysis of the marked regions of the mesoporous film by EDX reveals Zn/(In + Sn + Zn) ratios of 1.7% (dotted line) and 2.0% (solid line), respectively. These values are in good agreement with each other, but considerably lower than in single layer film (6.5%, see Section 1.3), meaning that less ZnO per volume of film was deposited in case of the multilayer film. This is probably caused by interplay between the transport of reactants (Zn^2+^ and/or O_2_) within the film and the conductivity of the mesoporous ITO. The transport leads to a favoured deposition in the top region, but is countered by the conductivity which causes a potential drop from the substrate towards the top of the film leading to favoured deposition in the bottom region of the film. In total these two effects antagonize each other and lead to an almost uniform deposition of ZnO, but to a lower extent than in single layer films.

#### 2.2.2. Influence of the Duration of ZnO Electrodeposition on DSSC Characteristics

**Figure 8.**
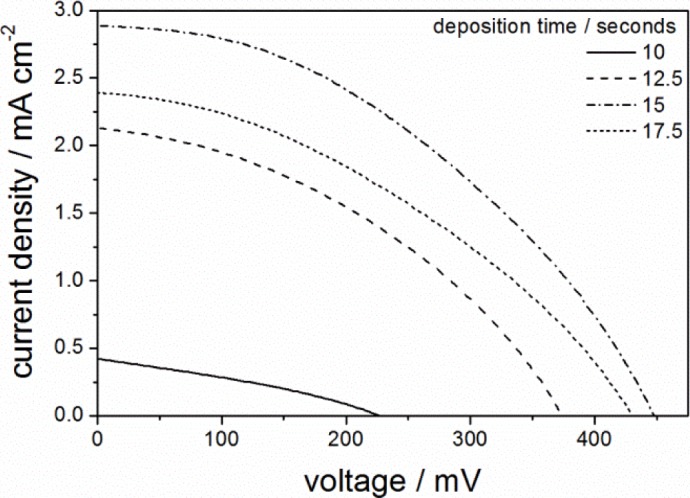
*J*-*V*-curves of D149-sensitized ZnO deposited into aged five-layer mesoporous ITO films prepared with PIB-PEO 20000 for different ZnO deposition times.

Given that aged single layered PIB-PEO 20000 templated ITO films showed the highest efficiency after 15 s of ZnO deposition, the deposition time was varied around this value also in case of the multilayer films. The resulting *J*-*V*-curves are shown in Figure 8. The fill factor is almost constant at ~0.4 and the solar to electrical power conversion efficiency η follows the same trend as the short-circuit current density *J*_SC_. As in case of the single layered film, pore blocking starts to show its effect for deposition times > 15 s. However, the photocurrent for 10 s ZnO deposition is still much lower than the maximum reached at 15 s, which is caused by the lower amount of ZnO electrodeposited per volume of film and unit of time. For the same reason, only after 15 s of ZnO deposition the multilayer film reaches almost the same *V*_OC_ value as that of single layer film. On the other hand, the larger overall film thickness and resulting higher total surface area of the multilayer film leads to a considerably higher *J*_SC_ value, so that the conversion efficiency η increases from 0.28% for the single layer film to 0.54% for the multilayer film. Note that the current density was only increased by a factor of 1.53, while the film thickness was increased by a factor of 2.75. This is most probably a consequence of the lower ZnO concentration in the multilayer film. However, longer ZnO deposition or faster ZnO deposition, e.g., by stirring the solution or applying a higher deposition potential, would not help in this issue. Thus, only larger pores or a different pore geometry would be expected to lead to further improvements of the system.

## 3. Experimental Section

A detailed description of the preparation of mesoporous ITO films can be found in the literatures [11,13]. In short, 442 mg In(III) acetylacetonate (Sigma Aldrich, 99, 99+%, St. Louis, MO, USA) were dissolved in 3 mL of MeOH/acetone (vol. 1:1) at 50 °C. Into the cooled down solution 35 mg Sn(IV) chloride (Sigma Aldrich, 99%, St. Louis, MO, USA) and 70 mg of the block copolymer poly(isobutylene)-b-poly(ethylene oxide) PIB-PEO 3000 or PIB-PEO 20000 (BASF SE, Ludwigshafen, Germany) were added. From the received sol thin films were produced in an EISA process (dip-coating) at a relative humidity of 18%–20% and a withdrawal rate of 10 mm s^−1^ on ITO glass (13–18 Ω/square, Visiontek Systems Ltd., Chester, UK). The obtained films were condensed at 300 °C for 12 h (~1 °C·min^−1^) and crystallized at 500 °C in an oven (10 °C·min^−1^). The slow condensation is needed to ensure homogeneous condensation of the inorganic precursors while the micells of the porogen are still in place. Thereby the subsequent crystallization at higher temperatures, removing the template and retaining the ITO mesostructure, leads to less mechanical stress caused by inhomogeneous crystallization seeds which would result in cracks. If the ramp was less steep or the calcination temperature of 500 °C was kept for a longer period of time or even chosen to be higher, films with bigger crystallites were obtained. This, on the one hand, leads to an increased electrical conductivity but, on the other hand, results in an inferior mesostructure because of pore blocking and collapsing pore walls. In order to prepare multilayered mesoporous ITO films each freshly dip-coated layer was heated to 200 °C within 30 min and kept at that temperature for another 30 min before dip-coating of another layer. After adding the final layer the removal of template (300 °C) and crystallization (500 °C) were achieved as mentioned above.

Before ZnO deposition, the mesoporous ITO films were either reduced by N_2_ in an oven for 2 h at 300 °C, aged for 100 days in air, or used less than one week after preparation (“as-prepared” films). ZnO electrodeposition was carried out from aqueous solution (DI water, 18.2 MΩ cm, Sartorius, Arium 611 DI, Goettingen, Germany) containing 5 mM ZnCl_2_ (Merck, 98%, Darmstadt, Germany) and 0.1 M KCl (Fluka, 99%, Sigma Aldrich, St. Louis, MO, USA) and saturated with O_2_ for 15 min prior to the start of the deposition, in a three-electrode setup (Amel Instruments model 7050 potentiostat, Milano, Italy) at −0.91 V *versus* an Ag/AgCl reference electrode (XR300, Radiometer Analytical, Villeurbanne, France, saturated aqueous AgCl/KCl solution) using a zinc wire as counter electrode. The solution was kept at a temperature of 70 °C and was not stirred, while the electrodeposition time was varied between 1.25 s and 10 s or 5 s and 20 s for mesoporous ITO films prepared with PIB-PEO 3000 or PIB-PEO 20000, respectively. The obtained films were dried at 120 °C, sensitized in a 0.5 mM solution of D149 dye in a mixture of acetonitrile and tert-butanol (1:1 by volume) with 1 mM cholic acid as co-adsorbent, and finally dried at 80 °C, each step lasting for 1 h.

In addition to X-ray diffraction and Kr adsorption measurements already discussed in the earlier study by von Graberg *et al.* [11], the mesoporous ITO films were characterized by scanning electron microscopy (SEM, Jeol JSM 6700 F FE, Akishima, Japan). Film characterization after 10 s of ZnO electrodeposition was performed using transmission electron microscopy (TEM) in combination with energy-dispersive X-ray spectroscopy (EDXS). The TEM (Jeol JEM-2100F-UHR, Akishima, Japan) was operated at 200 kV in scanning mode (STEM) to record high-angle annular dark-field (HAADF) micrographs. The samples were prepared by first polishing on polymer embedded diamond lapping films to a thickness of *ca*. 10 μm (Allied High Tech Multiprep, Compton, CA, USA) and then thinning out by Ar^+^ ion sputtering (Gatan 691 PIPS, Pleasanton, CA, USA) at 3 kV under small incident angles of 2° to 4° to a thickness of 30 to 70 nm. EDXS was measured using an Oxford Instruments INCA 200 (Oxford Instruments, Abingdon, UK) attached to the microscope. The sheet resistance of the films was measured by 4-point probing with a Keithley 193 system DMM (Cleveland, OH, USA), but note that these measurements were made at separate mesoporous ITO films deposited on non-conductive glass slides.

For the assembly of DSSCs, Pt-coated ITO/glass substrates were used as counter electrodes. The Pt was sputtered onto the counter electrodes for 120 s at 30 mA with an Argon pressure of 0.5 mbar by a Cressington sputter coater 108. Teflon tape with thickness of 100 μm and a circular cut-out with a diameter of 5 mm (to define the active area of the DSSC) was used as spacer between the two electrodes. The space between the electrodes was filled with a redox electrolyte containing 0.5 M tetrapropyl-ammoniumiodide and 0.05 M I_2_ in a mixture of acetonitrile and ethylene carbonate (1:4 by volume). Current-voltage curves (*J*-*V*-curves) were recorded using an IM6e electrochemical workstation (Zahner Elektrik, Kronach, Germany). Illumination of the DSSCs was carried out through the dye-sensitized film with simulated sunlight (100 mW·cm^−2^) from a Xe arc lamp filtered by an Oriel AM1.5D filter (Newport Corporation, Irvine, CA, USA).

## 4. Conclusions

The electrochemical deposition of ZnO in mesoporous ITO films for use in DSSCs is strongly influenced by the pore size and pore geometry; allowing in case of small or thin pores only the formation of a rather incomplete ZnO layer on the inner ITO pore walls before blocking the pore entrances. By increasing the pore size from 20–25 nm (PIB-PEO 3000) to 35–45 nm (PIB-PEO 20000), the ZnO deposition could be performed for a longer time enabling a higher coverage of the ITO surface by ZnO and leading to a higher open-circuit voltage *V*_OC_. Despite only of about 2.5% in thickness compared to common nanoporous ZnO films on dense ITO these films generate about 15% of their photocurrent density. Thus, a higher film thickness should lead to high conversion efficiencies. Multilayering the mesoporous ITO prepared with PIB-PEO 20000 led to an increase in photocurrent density *J*_SC_, however the necessary film thickness of several micrometers was not obtainable due instability of the layers in the subsequent dip-coating steps. If currently existing limits in sol-gel dip-coating of multilayered mesoporous ITO films can be overcome, it might be possible to further increase the number of layers per film and thereby the photocurrent density. However, a major loss due to back reaction of photogenerated electrons from bare ITO would still remain, making it necessary to increase the pore diameter even more in order to improve the coverage of the ITO by ZnO. Unfortunately, an amphiphilic block copolymer with a higher molecular weight, which could be used to form even larger pores, is not available at present; also the solubility of the copolymer in the ITO sol decreases drastically with increasing molecular weight. Only by replacing the soft templates by hard templates such as microspheres, it might be possible to create pores with the necessary size. It also has to be taken into account that larger pores will lead to a smaller surface area and eventually to a smaller amount of adsorbed dye and smaller photocurrent per volume of film, again limiting the achievable efficiency or making the formation of thicker films necessary.

Thus it must be concluded, that the use of ordered mesoporous TCO films modified by electrodeposition of metal oxides in DSSC is currently limited by the realizable pore sizes and geometries and the low thickness of the films.

## Supplementary Materials

Supplementary File 1
