# Supplementary material for: Limits of ZnO Electrodeposition in Mesoporous Tin Doped Indium Oxide Films in View of Application in Dye-Sensitized Solar Cells
Source: Materials (Basel). 2014 Apr 23;7(4):3291–304. doi: 10.3390/ma7043291 (PMC5453342; doi:10.3390/ma7043291)

Supplementary

Figure S1. UV-vis spectra in transmission of the ITO substrate and mesoporous ITO films templated with the block copolymers PIB-PEO 3000 and PIB-PEO 20000. The spectra were recorded with a Varian Cary 4000.

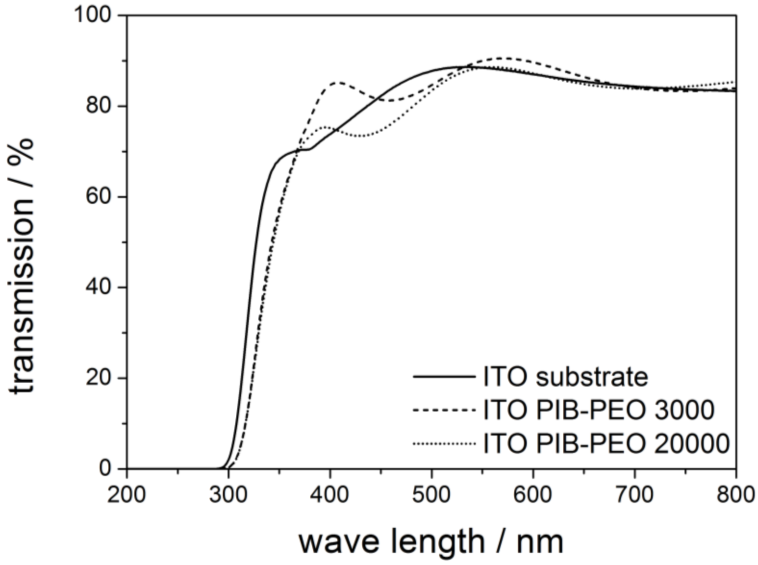

Supplement: Supplementary File 1 [file materials-07-03291-s001.pdf]
